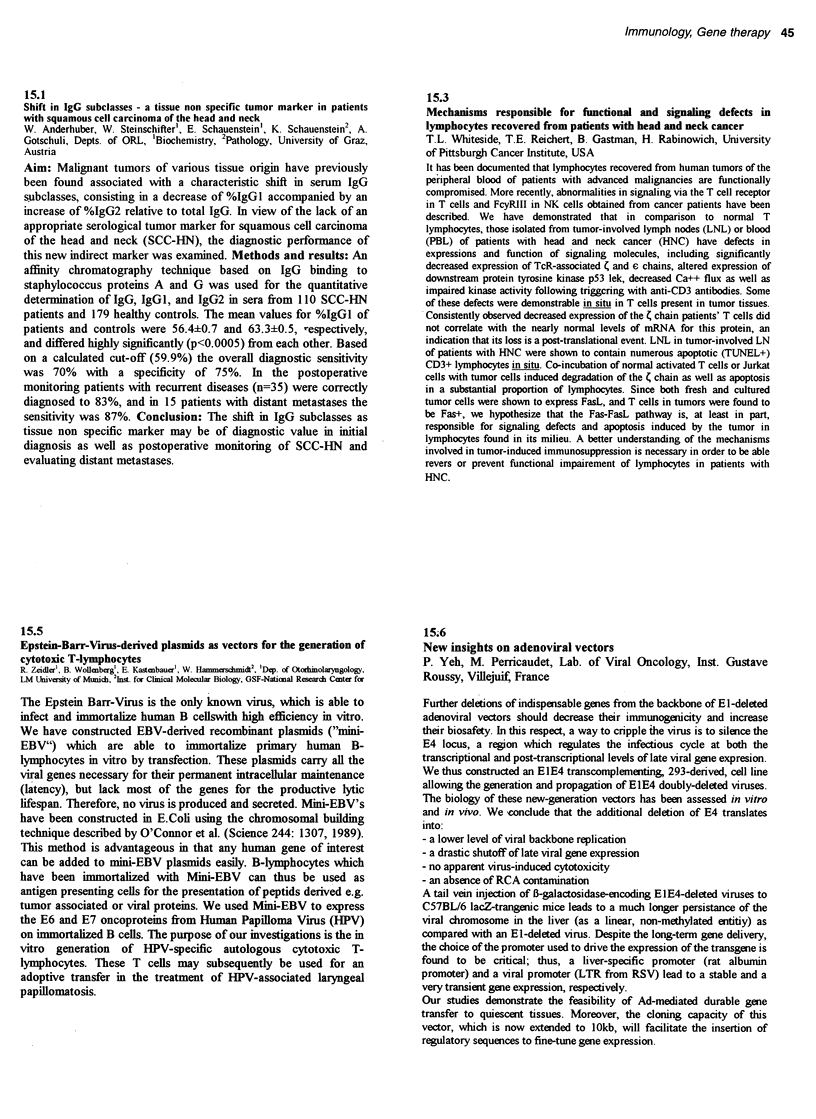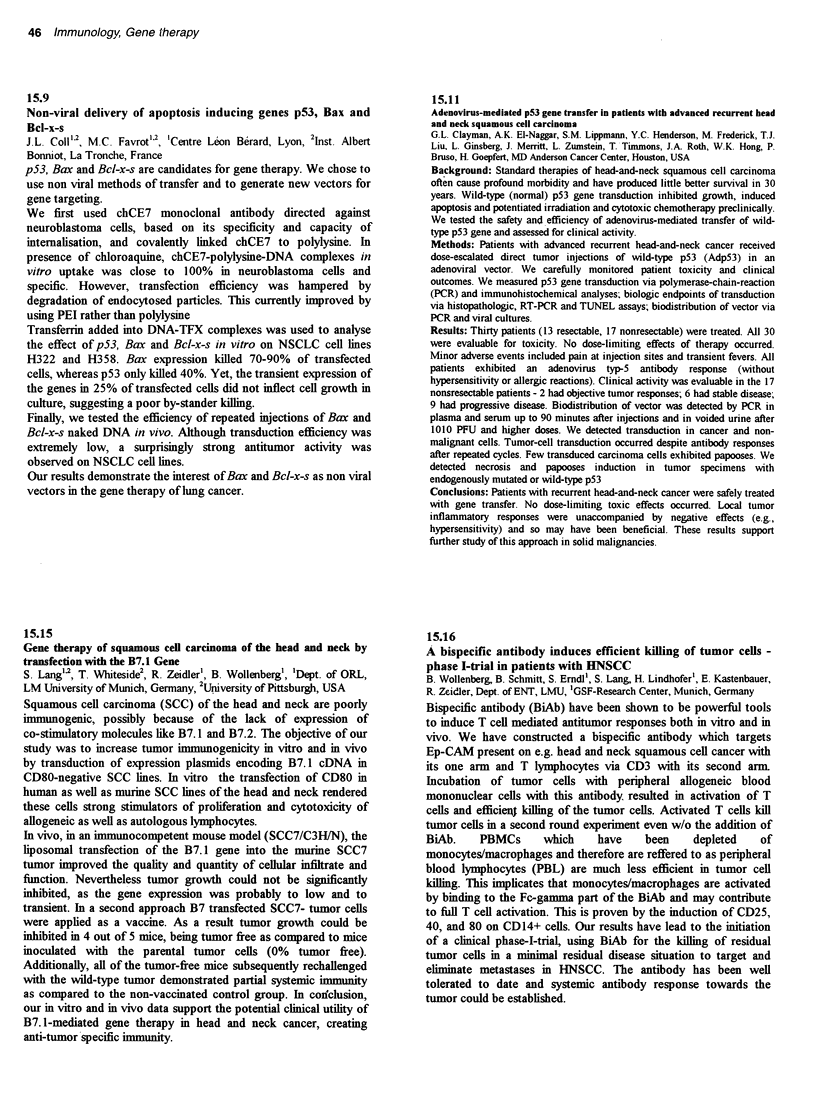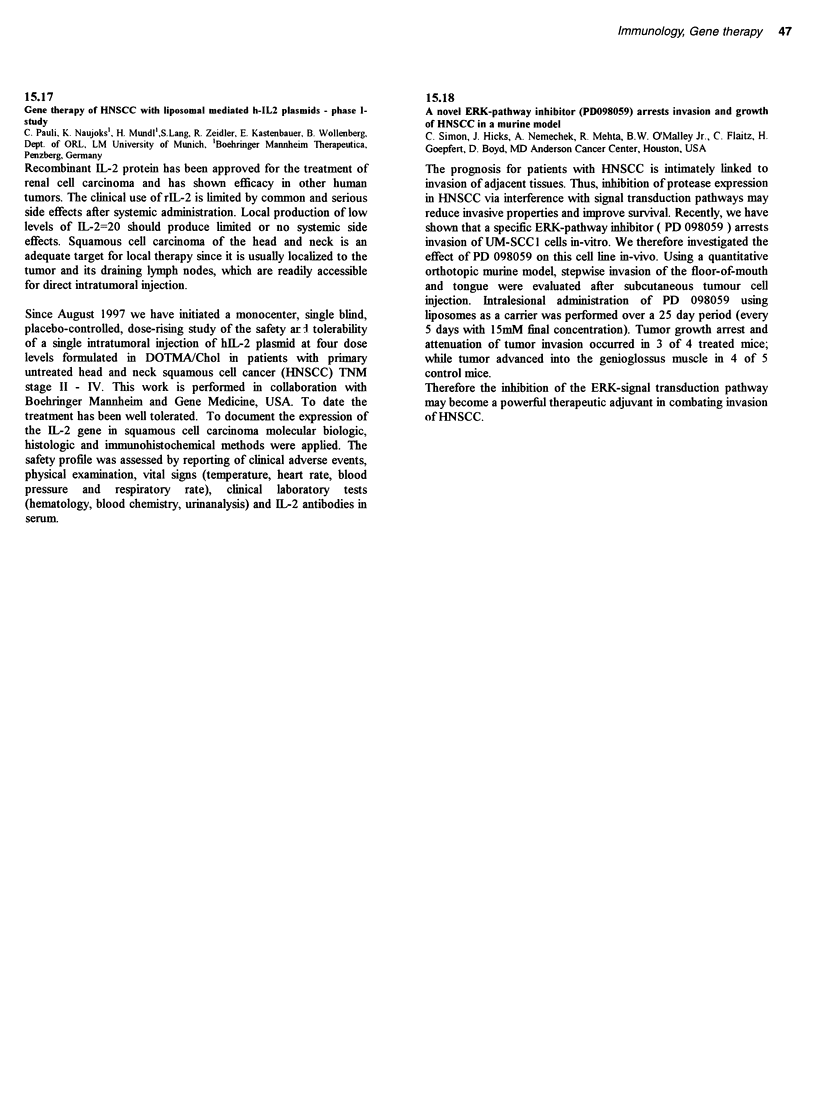# Immunology, Gene therapy

**Published:** 1998

**Authors:** 


**Shift in IgG subclasses - a tissue non specific tumor marker in patients with squamous cell carcinoma of the head and neck**

W. Anderhuber, W. Steinschifter[1], E. Schauenstein[1], K. Schauenstein[2], A. Gotschuli, Depts. of ORL, [1]Biochemistry, [2]Pathology, University of Graz, Austria

**Aim:** Malignant tumors of various tissue origin have previously been found associated with a characteristic shift in serum IgG subclasses, consisting in a decrease of %IgG1 accompanied by an increase of %IgG2 relative to total IgG. In view of the lack of an appropriate serological tumor marker for squamous cell carcinoma of the head and neck (SCC-HN), the diagnostic performance of this new indirect marker was examined. **Methods and results:** An affinity chromatography technique based on IgG binding to staphylococcus proteins A and G was used for the quantitative determination of IgG, IgG1, and IgG2 in sera from 110 SCC-HN patients and 179 healthy controls. The mean values for %IgG1 of patients and controls were 56.4±0.7 and 63.3±0.5, respectively, and differed highly significantly ($p<0.0005$) from each other. Based on a calculated cut-off (59.9%) the overall diagnostic sensitivity was 70% with a specificity of 75%. In the postoperative monitoring patients with recurrent diseases (n=35) were correctly diagnosed to 83%, and in 15 patients with distant metastases the sensitivity was 87%. **Conclusion:** The shift in IgG subclasses as tissue non specific marker may be of diagnostic value in initial diagnosis as well as postoperative monitoring of SCC-HN and evaluating distant metastases.

### 15.3

**Mechanisms responsible for functional and signaling defects in lymphocytes recovered from patients with head and neck cancer**

T.L. Whiteside, T.E. Reichert, B. Gastman, H. Rabinowich, University of Pittsburgh Cancer Institute, USA

It has been documented that lymphocytes recovered from human tumors of the peripheral blood of patients with advanced malignancies are functionally compromised. More recently, abnormalities in signaling via the T cell receptor in T cells and FcγRIII in NK cells obtained from cancer patients have been described. We have demonstrated that in comparison to normal T lymphocytes, those isolated from tumor-involved lymph nodes (LNL) or blood (PBL) of patients with head and neck cancer (HNC) have defects in expressions and function of signaling molecules, including significantly decreased expression of TcR-associated ζ and ε chains, altered expression of downstream protein tyrosine kinase p53 lck, decreased Ca++ flux as well as impaired kinase activity following triggering with anti-CD3 antibodies. Some of these defects were demonstrable in situ in T cells present in tumor tissues. Consistently observed decreased expression of the ζ chain patients' T cells did not correlate with the nearly normal levels of mRNA for this protein, an indication that its loss is a post-translational event. LNL in tumor-involved LN of patients with HNC were shown to contain numerous apoptotic (TUNEL+) CD3+ lymphocytes in situ. Co-incubation of normal activated T cells or Jurkat cells with tumor cells induced degradation of the ζ chain as well as apoptosis in a substantial proportion of lymphocytes. Since both fresh and cultured tumor cells were shown to express FasL, and T cells in tumors were found to be Fas+, we hypothesize that the Fas-FasL pathway is, at least in part, responsible for signaling defects and apoptosis induced by the tumor in lymphocytes found in its milieu. A better understanding of the mechanisms involved in tumor-induced immunosuppression is necessary in order to be able revers or prevent functional impairement of lymphocytes in patients with HNC.

### 15.5

**Epstein-Barr-Virus-derived plasmids as vectors for the generation of cytotoxic T-lymphocytes**

R. Zeidler[1], B. Wollenberg[1], E. Kastenbauer[1], W. Hammerschmidt[2], [1]Dep. of Otorhinolaryngology, LM University of Munich, [2]Inst. for Clinical Molecular Biology, GSF-National Research Center for

The Epstein Barr-Virus is the only known virus, which is able to infect and immortalize human B cellswith high efficiency in vitro. We have constructed EBV-derived recombinant plasmids ("mini-EBV") which are able to immortalize primary human B-lymphocytes in vitro by transfection. These plasmids carry all the viral genes necessary for their permanent intracellular maintenance (latency), but lack most of the genes for the productive lytic lifespan. Therefore, no virus is produced and secreted. Mini-EBV's have been constructed in E.Coli using the chromosomal building technique described by O'Connor et al. (Science 244: 1307, 1989). This method is advantageous in that any human gene of interest can be added to mini-EBV plasmids easily. B-lymphocytes which have been immortalized with Mini-EBV can thus be used as antigen presenting cells for the presentation of peptids derived e.g. tumor associated or viral proteins. We used Mini-EBV to express the E6 and E7 oncoproteins from Human Papilloma Virus (HPV) on immortalized B cells. The purpose of our investigations is the in vitro generation of HPV-specific autologous cytotoxic T-lymphocytes. These T cells may subsequently be used for an adoptive transfer in the treatment of HPV-associated laryngeal papillomatosis.

### 15.6

**New insights on adenoviral vectors**

P. Yeh, M. Perricaudet, Lab. of Viral Oncology, Inst. Gustave Roussy, Villejuif, France

Further deletions of indispensable genes from the backbone of E1-deleted adenoviral vectors should decrease their immunogenicity and increase their biosafety. In this respect, a way to cripple the virus is to silence the E4 locus, a region which regulates the infectious cycle at both the transcriptional and post-transcriptional levels of late viral gene expresion. We thus constructed an E1E4 transcomplementing, 293-derived, cell line allowing the generation and propagation of E1E4 doubly-deleted viruses. The biology of these new-generation vectors has been assessed *in vitro* and *in vivo*. We conclude that the additional deletion of E4 translates into:

- a lower level of viral backbone replication
- a drastic shutoff of late viral gene expression
- no apparent virus-induced cytotoxicity
- an absence of RCA contamination

A tail vein injection of ß-galactosidase-encoding E1E4-deleted viruses to C57BL/6 lacZ-trangenic mice leads to a much longer persistance of the viral chromosome in the liver (as a linear, non-methylated entitiy) as compared with an E1-deleted virus. Despite the long-term gene delivery, the choice of the promoter used to drive the expression of the transgene is found to be critical; thus, a liver-specific promoter (rat albumin promoter) and a viral promoter (LTR from RSV) lead to a stable and a very transient gene expression, respectively.

Our studies demonstrate the feasibility of Ad-mediated durable gene transfer to quiescent tissues. Moreover, the cloning capacity of this vector, which is now extended to 10kb, will facilitate the insertion of regulatory sequences to fine-tune gene expression.

### 15.9

### Non-viral delivery of apoptosis inducing genes p53, Bax and Bcl-x-s

J.L. Coll[1,2], M.C. Favrot[1,2], [1]Centre Léon Bérard, Lyon, [2]Inst. Albert Bonniot, La Tronche, France

*p53, Bax* and *Bcl-x-s* are candidates for gene therapy. We chose to use non viral methods of transfer and to generate new vectors for gene targeting.

We first used chCE7 monoclonal antibody directed against neuroblastoma cells, based on its specificity and capacity of internalisation, and covalently linked chCE7 to polylysine. In presence of chloroaquine, chCE7-polylysine-DNA complexes *in vitro* uptake was close to 100% in neuroblastoma cells and specific. However, transfection efficiency was hampered by degradation of endocytosed particles. This currently improved by using PEI rather than polylysine

Transferrin added into DNA-TFX complexes was used to analyse the effect of *p53, Bax* and *Bcl-x-s in vitro* on NSCLC cell lines H322 and H358. *Bax* expression killed 70-90% of transfected cells, whereas p53 only killed 40%. Yet, the transient expression of the genes in 25% of transfected cells did not inflect cell growth in culture, suggesting a poor by-stander killing.

Finally, we tested the efficiency of repeated injections of *Bax* and *Bcl-x-s* naked DNA *in vivo*. Although transduction efficiency was extremely low, a surprisingly strong antitumor activity was observed on NSCLC cell lines.

Our results demonstrate the interest of *Bax* and *Bcl-x-s* as non viral vectors in the gene therapy of lung cancer.

### 15.11

### Adenovirus-mediated p53 gene transfer in patients with advanced recurrent head and neck squamous cell carcinoma

G.L. Clayman, A.K. El-Naggar, S.M. Lippmann, Y.C. Henderson, M. Frederick, T.J. Liu, L. Ginsberg, J. Merritt, L. Zumstein, T. Timmons, J.A. Roth, W.K. Hong, P. Bruso, H. Goepfert, MD Anderson Cancer Center, Houston, USA

**Background:** Standard therapies of head-and-neck squamous cell carcinoma often cause profound morbidity and have produced little better survival in 30 years. Wild-type (normal) p53 gene transduction inhibited growth, induced apoptosis and potentiated irradiation and cytotoxic chemotherapy preclinically. We tested the safety and efficiency of adenovirus-mediated transfer of wild-type p53 gene and assessed for clinical activity.

**Methods:** Patients with advanced recurrent head-and-neck cancer received dose-escalated direct tumor injections of wild-type p53 (Adp53) in an adenoviral vector. We carefully monitored patient toxicity and clinical outcomes. We measured p53 gene transduction via polymerase-chain-reaction (PCR) and immunohistochemical analyses; biologic endpoints of transduction via histopathologic, RT-PCR and TUNEL assays; biodistribution of vector via PCR and viral cultures.

**Results:** Thirty patients (13 resectable, 17 nonresectable) were treated. All 30 were evaluable for toxicity. No dose-limiting effects of therapy occurred. Minor adverse events included pain at injection sites and transient fevers. All patients exhibited an adenovirus typ-5 antibody response (without hypersensitivity or allergic reactions). Clinical activity was evaluable in the 17 nonsresectable patients - 2 had objective tumor responses; 6 had stable disease; 9 had progressive disease. Biodistribution of vector was detected by PCR in plasma and serum up to 90 minutes after injections and in voided urine after 1010 PFU and higher doses. We detected transduction in cancer and non-malignant cells. Tumor-cell transduction occurred despite antibody responses after repeated cycles. Few transduced carcinoma cells exhibited papooses. We detected necrosis and papooses induction in tumor specimens with endogenously mutated or wild-type p53

**Conclusions:** Patients with recurrent head-and-neck cancer were safely treated with gene transfer. No dose-limiting toxic effects occurred. Local tumor inflammatory responses were unaccompanied by negative effects (e.g., hypersensitivity) and so may have been beneficial. These results support further study of this approach in solid malignancies.

### 15.15

### Gene therapy of squamous cell carcinoma of the head and neck by transfection with the B7.1 Gene

S. Lang[1,2], T. Whiteside[2], R. Zeidler[1], B. Wollenberg[1], [1]Dept. of ORL, LM University of Munich, Germany, [2]University of Pittsburgh, USA

Squamous cell carcinoma (SCC) of the head and neck are poorly immunogenic, possibly because of the lack of expression of co-stimulatory molecules like B7.1 and B7.2. The objective of our study was to increase tumor immunogenicity in vitro and in vivo by transduction of expression plasmids encoding B7.1 cDNA in CD80-negative SCC lines. In vitro the transfection of CD80 in human as well as murine SCC lines of the head and neck rendered these cells strong stimulators of proliferation and cytotoxicity of allogeneic as well as autologous lymphocytes.

In vivo, in an immunocompetent mouse model (SCC7/C3H/N), the liposomal transfection of the B7.1 gene into the murine SCC7 tumor improved the quality and quantity of cellular infiltrate and function. Nevertheless tumor growth could not be significantly inhibited, as the gene expression was probably to low and to transient. In a second approach B7 transfected SCC7- tumor cells were applied as a vaccine. As a result tumor growth could be inhibited in 4 out of 5 mice, being tumor free as compared to mice inoculated with the parental tumor cells (0% tumor free). Additionally, all of the tumor-free mice subsequently rechallenged with the wild-type tumor demonstrated partial systemic immunity as compared to the non-vaccinated control group. In conclusion, our in vitro and in vivo data support the potential clinical utility of B7.1-mediated gene therapy in head and neck cancer, creating anti-tumor specific immunity.

### 15.16

### A bispecific antibody induces efficient killing of tumor cells - phase I-trial in patients with HNSCC

B. Wollenberg, B. Schmitt, S. Erndl[1], S. Lang, H. Lindhofer[1], E. Kastenbauer, R. Zeidler, Dept. of ENT, LMU, [1]GSF-Research Center, Munich, Germany

Bispecific antibody (BiAb) have been shown to be powerful tools to induce T cell mediated antitumor responses both in vitro and in vivo. We have constructed a bispecific antibody which targets Ep-CAM present on e.g. head and neck squamous cell cancer with its one arm and T lymphocytes via CD3 with its second arm. Incubation of tumor cells with peripheral allogeneic blood mononuclear cells with this antibody. resulted in activation of T cells and efficient killing of the tumor cells. Activated T cells kill tumor cells in a second round experiment even w/o the addition of BiAb. PBMCs which have been depleted of monocytes/macrophages and therefore are reffered to as peripheral blood lymphocytes (PBL) are much less efficient in tumor cell killing. This implicates that monocytes/macrophages are activated by binding to the Fc-gamma part of the BiAb and may contribute to full T cell activation. This is proven by the induction of CD25, 40, and 80 on CD14+ cells. Our results have lead to the initiation of a clinical phase-I-trial, using BiAb for the killing of residual tumor cells in a minimal residual disease situation to target and eliminate metastases in HNSCC. The antibody has been well tolerated to date and systemic antibody response towards the tumor could be established.

### 15.17

**Gene therapy of HNSCC with liposomal mediated h-IL2 plasmids - phase I-study**

C. Pauli, K. Naujoks[1], H. Mundl[1], S.Lang, R. Zeidler, E. Kastenbauer, B. Wollenberg, Dept. of ORL, LM University of Munich, [1]Boehringer Mannheim Therapeutica, Penzberg, Germany

Recombinant IL-2 protein has been approved for the treatment of renal cell carcinoma and has shown efficacy in other human tumors. The clinical use of rIL-2 is limited by common and serious side effects after systemic administration. Local production of low levels of IL-2=20 should produce limited or no systemic side effects. Squamous cell carcinoma of the head and neck is an adequate target for local therapy since it is usually localized to the tumor and its draining lymph nodes, which are readily accessible for direct intratumoral injection.

Since August 1997 we have initiated a monocenter, single blind, placebo-controlled, dose-rising study of the safety and tolerability of a single intratumoral injection of hIL-2 plasmid at four dose levels formulated in DOTMA/Chol in patients with primary untreated head and neck squamous cell cancer (HNSCC) TNM stage II - IV. This work is performed in collaboration with Boehringer Mannheim and Gene Medicine, USA. To date the treatment has been well tolerated. To document the expression of the IL-2 gene in squamous cell carcinoma molecular biologic, histologic and immunohistochemical methods were applied. The safety profile was assessed by reporting of clinical adverse events, physical examination, vital signs (temperature, heart rate, blood pressure and respiratory rate), clinical laboratory tests (hematology, blood chemistry, urinanalysis) and IL-2 antibodies in serum.

### 15.18

**A novel ERK-pathway inhibitor (PD098059) arrests invasion and growth of HNSCC in a murine model**

C. Simon, J. Hicks, A. Nemechek, R. Mehta, B.W. O'Malley Jr., C. Flaitz, H. Goepfert, D. Boyd, MD Anderson Cancer Center, Houston, USA

The prognosis for patients with HNSCC is intimately linked to invasion of adjacent tissues. Thus, inhibition of protease expression in HNSCC via interference with signal transduction pathways may reduce invasive properties and improve survival. Recently, we have shown that a specific ERK-pathway inhibitor ( PD 098059 ) arrests invasion of UM-SCC1 cells in-vitro. We therefore investigated the effect of PD 098059 on this cell line in-vivo. Using a quantitative orthotopic murine model, stepwise invasion of the floor-of-mouth and tongue were evaluated after subcutaneous tumour cell injection. Intralesional administration of PD 098059 using liposomes as a carrier was performed over a 25 day period (every 5 days with 15mM final concentration). Tumor growth arrest and attenuation of tumor invasion occurred in 3 of 4 treated mice; while tumor advanced into the genioglossus muscle in 4 of 5 control mice.

Therefore the inhibition of the ERK-signal transduction pathway may become a powerful therapeutic adjuvant in combating invasion of HNSCC.